# Management of tethered cord syndrome in adults: a case report in Cameroon

**DOI:** 10.11604/pamj.2014.17.217.3516

**Published:** 2014-03-19

**Authors:** Mathieu Motah, Felix Uduma, Aurélien Ndoumbe, Mireille Georgette Moumi, Vincent de Paul Djientcheu

**Affiliations:** 1 Neurosurgical Unit, Department of Surgery University of Douala, Cameroon; 2 Departmentof Radiology, Faculty of Clinical Sciences, College Of Health Sciences, University Of Uyo, Nigeria; 3 Neurosurgical Unit, of Surgery, University of Yaoundé 1, Yaoundé. Cameroon

**Keywords:** Tethered cord syndrome, adult, surgery

## Abstract

Tethered cord syndrome (TCS) is spinal cord fixation from multiple pathological entities. No case of TCShas been reported in our region. The goal of this case report was to describe a TCS managed at the Douala General hospital. Mrs. EEL, 23 year old consulted in 2012 for urinary and fecal incontinence. She had a past history of a spina bifida at birth operated on day two of life. On admission, lumbar MRI showed an abnormally low lying conus medullaris ending at S. Microsurgery permitted to gradually detach the spinal cord from subcutaneous tissue and carefully free the spinal nerves. A 12 months post-surgery, the patient could control defecation, and achieve proper micturition. TCS should also be ruled out in patients who present with urinary and ano-rectal symptoms especially of childhood onset; more so with present day availability of modern radiological tools like MRI.

## Introduction

Tethered cord syndrome (TCS) is spinal cord fixation from multiple pathological entities. The consequence is hindrance of normal physiological motion of this component of the central nervous system. Most of the conditions resulting in TCS are congenital. The acquired causes of TCS are as a result of spinal injury or following a myelomeningocele surgery [1]. The incidence of neural tube defect in Cameroon is 1. 99 per 1000 births. [2]. Surgery of repairing myelomeningocele consist of local closure alone or local closure with associated CSF shunting. The surgical complications of myelomeningocele are wound dehiscence (13. 69%), shunt infection (1. 37%), meningitis (1. 37%) and iatrogenic pulmonary edema (1. 37%) [1, 3]. So far; no case of TCS has been reported in our region. The goal of this case report is to describe the diagnosis, treatment and outcome of a TCS case followed up at the Doula General hospital, Cameroon.

## Patient and observation

Mrs. EEL, a 23 year old lady came for consultation on the 20th January 2012 on account of urinary and fecal incontinence. She was born through caesarian section at Douala with a spina bifida associated with club feet. She was operated for spina bifida by a General surgeon on second day of life. Thereafter, she has been having urinary and fecal incontinence. On physical examination, she had a height of 150cm, a limping gait, dysgenesis of toes and club feet. The muscle power of both lower limbs was 3/5. There was hypoesthesia of the perineum and major labia. The ano-rectal reflex was indifferent. Other physical examinations were normal.

**Figure 1:**
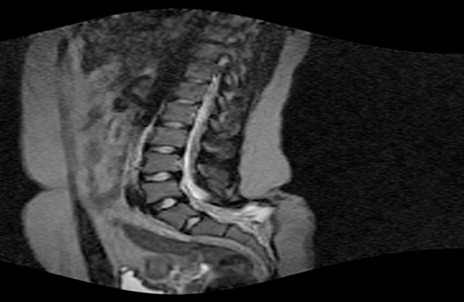
Lumbar MRI showing a lowlying conus medullaris at S1and filum terminale fixed to subcutaneous

Conventional radiographs showed spina bifida at L5, S1, S2, S3. Lumbar MRI showed an abnormally low lying conus medullaris ending at S1. A thick filum terminale attached the spinal cord to the subcutaneous fat (Figure 1). Patient and family consented to surgery after the explanation of surgical benefits of symptomatology amelioration. Surgery was done on the 28/02/2012.

**Figure 2:**
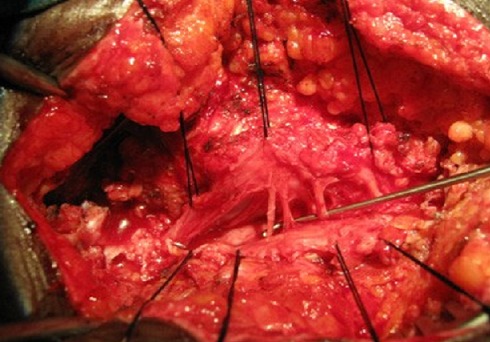
Intra operative picture showing a low-lying spinal cord and spinal roots fixed to fatty tissue

**Operative technique:** The patient was placed in a prone position with the head resting on a doughnut-shaped headrest. Abdominal compression was prevented with foam rubber bolsters placed underneath patient's chest and abdomen. Under general anaesthesia, we made a long midline incision following the spinous processes from L4 to S4 with a plain knife Haemostasis of the sub- dermal layers and other ensuing areas was achieved using a warm compression, aspiration and electro coagulation. A section of the fascia of the spinal muscles on the spinous crest of L4 followed by separation of the spinal muscles from the vertebral arches was done. Laminectomy of L4 was carried out and this helped us to localize the normal dural margins. The dura mater was linearly opened from L4 to S4 and suspended with silk sutures N°3/0. Intra-operative observation was a cord fixed to subcutaneous tissue and a thick filum terminale extending to S1 (Figure 2). The usage of WFNS neurosurgical microscope permitted us to gradually detach the spinal cord from subcutaneous tissue and carefully free the spinal nerves. The spinal cord was then re-inserted into the vertebral canal. Sequential closure of dura mater with silk suture N° 5/0, repositioning of para- vertebral muscles with polylactine910 suture N° 3/0 and skin with polyamide 6 suture N°1 were done.

**Outcome:** Postoperative cerebrospinal fluid leakage without complicating infections occurred but was reinforced with sutures. The patient was placed in a prone position until scaring of the wound occurred by the 4th week. She was subsequently sent for physiotherapy to regain lower limb and perineal motor activity.

Re-evaluation on 28 February 2013 (12 months post-surgery) showed that the patient could control defecation, had achieved good micturition without diapers or catheters and had lower limb muscle strength of 5/5.

## Discussion

**Clinical presentation:** The common clinical presentations of myelomeningocele and TCS are interwoven. These include neurogenic bladder (with development of primary or secondary incontinence), leg or foot weakness and foot deformity [1, 4]. Our patient presented with club feet and urinary incontinence. We think these abnormalities already diagnosed at birth persisted with the presence of TCS despite previous neonatal repair of spina bifida.

**Assessment:** Ideally, spinal cord tension and elasticity could be measured and assessed in an objective manner. That is not technically possible in a non invasive clinical manner. Therefore, we must rely on more indirect measures such as cord position with magnetic resonance imaging (MRI) [1, 5]. In our case the MRI permitted us to make a diagnosis of TCS, given that the filum terminale was found at S1, whereas in a 23 year old, it should be found at L2. A pelvic ultrasonography which was not done would have allowed us to appreciate the bladder dimensions and identify any ano-rectal malformation as advocated by certain authors [5].

**Surgical decision making:** The natural history of low-lying conus medullaris from a fatty infiltration and thickened filum terminale is now accepted to be acause, of progressive loss of neurological functions usually over years. This knowledge together with the low risk of surgical intervention and high likelihood of cure with a single procedure justify operation [1, 5, 6]. In the present case, the surgery permitted us to detach the filum terminale attached to the sub cutaneous fat and to free the lumbar and sacral nerves.

**Outcome:** Our 12-monrh post-operative patient's re-evaluation was favorable with good urinary/ fecal continence and reinstatement of lower limb muscle tone in keeping with high potential of cure in a single surgical procedure.

## Conclusion

Tethered cord syndrome should also be ruled out in patients who present with urinary and ano-rectal symptoms especially of childhood onset. More sowith present day availability of modern radiological tools like MRI
